# A Novel Approach to Characterize the Lipidome of Marine Archaeon *Nitrosopumilus maritimus* by Ion Mobility Mass Spectrometry

**DOI:** 10.3389/fmicb.2021.735878

**Published:** 2021-12-02

**Authors:** Kai P. Law, Wei He, Jianchang Tao, Chuanlun Zhang

**Affiliations:** ^1^Southern University of Science and Technology, SUSTech Academy for Advanced Interdisciplinary Studies, Shenzhen, China; ^2^Shenzhen Key Laboratory of Marine Archaea Geo-Omics, Southern University of Science and Technology, Shenzhen, China; ^3^Department of Ocean Science and Engineering, Southern University of Science and Technology, Shenzhen, China; ^4^Southern Marine Science and Engineering Guangdong Laboratory (Guangzhou), Guangzhou, China

**Keywords:** archaea, *thaumarchaeota*, *Nitrosopumilus maritimus*, lipidomics, ion mobility mass spectrometry

## Abstract

Archaea are differentiated from the other two domains of life by their biomolecular characteristics. One such characteristic is the unique structure and composition of their lipids. Characterization of the whole set of lipids in a biological system (the lipidome) remains technologically challenging. This is because the lipidome is innately complex, and not all lipid species are extractable, separable, or ionizable by a single analytical method. Furthermore, lipids are structurally and chemically diverse. Many lipids are isobaric or isomeric and often indistinguishable by the measurement of mass or even their fragmentation spectra. Here we developed a novel analytical protocol based on liquid chromatography ion mobility mass spectrometry to enhance the coverage of the lipidome and characterize the conformations of archaeal lipids by their collision cross-sections (CCSs). The measurements of ion mobility revealed the gas-phase ion chemistry of representative archaeal lipids and provided further insights into their attributions to the adaptability of archaea to environmental stresses. A comprehensive characterization of the lipidome of mesophilic marine thaumarchaeon, *Nitrosopumilus maritimus* (strain SCM1) revealed potentially an unreported phosphate- and sulfate-containing lipid candidate by negative ionization analysis. It was the first time that experimentally derived CCS values of archaeal lipids were reported. Discrimination of crenarchaeol and its proposed stereoisomer was, however, not achieved with the resolving power of the SYNAPT G2 ion mobility system, and a high-resolution ion mobility system may be required for future work. Structural and spectral libraries of archaeal lipids were constructed in non-vendor-specific formats and are being made available to the community to promote research of Archaea by lipidomics.

## Introduction

Lipids are conventionally defined as organic molecules insoluble in water, but highly soluble in organic solvents. However, there are examples of lipids that do not adhere to this rudimentary definition. Biogenic lipids are now defined based on their biosynthetic origin and chemical structures (Brown and Murphy, 2009) as hydrophobic or amphiphilic small molecules that originate entirely or in part from two different types of biochemical building blocks: Ketoacyl groups and isoprene units (Fahy et al., 2005, 2009). The former categorize diverse classes of lipids that contain fatty acyl chains, whereas the latter cover all lipid species identified in Archaea as well as several species in the Bacteria and Eukarya (Brown and Murphy, 2009). Based on the chemical composition, lipids are classified by this system into eight major categories: fatty acyls, glycerolipids, glycerophospholipids, sphingolipids, sterols, prenols, saccharolipids (glycolipids), and polyketides, each of which is subdivided into classes and subclasses.

The structural diversity of lipids results in a range of physicochemical properties essential for their functions in biological processes, including structural components of cell membranes (Van Meer et al., 2008), energy storage molecules (Nakamura et al., 2014), signaling molecules (Wymann and Schneiter, 2008; Rangholia et al., 2021), protein recruitment platforms (Saliba et al., 2015) and substrates for post-translational protein-lipid modification (Resh, 2016; Eichler and Guan, 2017). While the structural or chemical diversity confers specific properties on lipids, the compositional diversity of lipids in a biological system affects the collective behavior of lipids in membranes (Harayama and Riezman, 2018). Due to their amphiphilic nature and their near cylindrical shape, glycerophospholipids are the main components of cellular membranes in the three domains of life (López-Lara and Geiger, 2017). Nevertheless, physicochemical properties of membrane glycerophospholipids vary significantly between the domains of organisms. The differences between bacterial/eukaryotic and archaeal lipids are thought to enable Archaea to survive in inhospitable environments (Oger and Cario, 2013) and are the basis of the “lipid divide” (Koga, 2011; Exterkate et al., 2021; Sohlenkamp, 2021). Comprehensive characterization of the lipidome of archaea and the genetically modified or synthetic organisms (Villanueva et al., 2020; Exterkate et al., 2021) may provide us insights into an enigma of microbial evolution assisted by membrane functions (Villanueva et al., 2017).

*Thaumarchaeota* (Brochier-Armanet et al., 2008) were initially observed in temperate marine environments and classified as a sister group of hyperthermophilic *Crenarchaeota* (DeLong, 1992; Fuhrman et al., 1992). Subsequent studies showed that *Thaumarchaeota* are widespread and abundant across a great variety of ecosystems (Schleper and Nicol, 2010; Stahl and Torre, 2012; Stieglmeier et al., 2014). The two major groups of *Thaumarchaeota* are group I.1a that encompass mainly sequences from marine habitats and group I.1b that contain sequences mainly from freshwater or soil habitats (DeLong, 1998). Phylogenomic analysis suggested that *Thaumarchaeota* evolved from geothermal environments and gradually migrated into mesophilic soil before diversifying into marine settings (Yang et al., 2021). A distinctive feature of *Thaumarchaeota* is that the majority of them grow chemolithoautotrophically and gain energy by aerobic oxidization of ammonia, urea, or cyanate to nitrite (Könneke et al., 2005; Schleper and Nicol, 2010; Alonso-Sáez et al., 2012; Stahl and Torre, 2012; Tolar et al., 2017; Kitzinger et al., 2019). The high abundance of *Thaumarchaeota* in many different environments has led to the proposition that they play a critical role in the global nitrogen and carbon cycles (Schleper and Nicol, 2010; Stahl and Torre, 2012). *Nitrosopumilus maritimus* (strain SCM1) was the first isolated representative of planktonic *Thaumarchaeota* (Könneke et al., 2005) with its genome sequenced in 2010 (Walker et al., 2010). It has since become a model organism of chemoautotrophic *Thaumarchaeota* for the studies of their specific metabolism and physiological responses.

**FIGURE 1:**
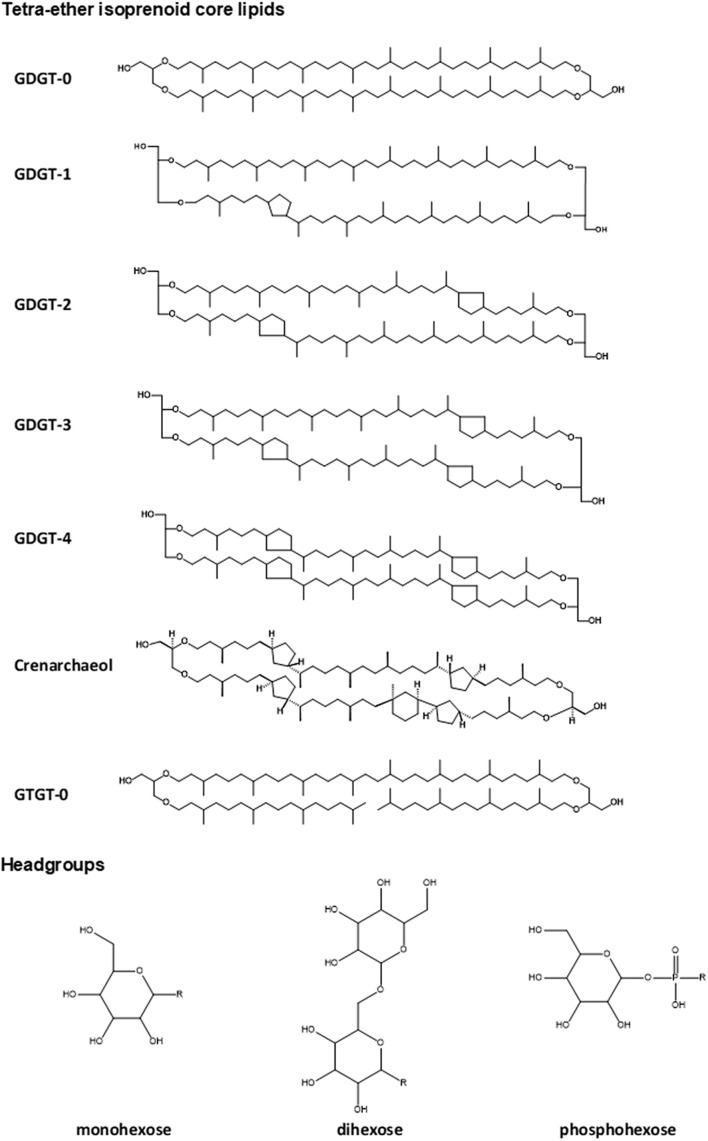
Structures of common lipids found in *N. maritimus*. These lipids are typically composed of a diether or tetraether isoprenoid core with one or two headgroups linked to the terminal hydroxyl moieties. Correct stereochemical form was drawn for crenarchaeol (as it is a unique GDGT compound) but not for all other compounds to avoid exhaustive use of space.

The lipidome of *N. maritimus* was first characterized by high-performance liquid chromatography-mass spectrometry (HPLC-MS), which detected the presence of glycerol dialkyl glycerol tetraethers (GDGTs) with 0–4 cyclopentane rings, a unique lipid marker, crenarchaeol, and glycerol trialkyl glycerol tetraethers (GTGTs). The intact polar lipids of these compounds consist of monohexose, dihexose, and phosphohexose (Schouten et al., 2008; Figure 1). A more sophisticated examination of the lipid composition of *N. maritimus* cultures was performed using reverse-phase liquid chromatography coupled to a high-resolution quadrupole time-of-flight mass spectrometer (RPLC-HR-QToF-MS), which identified a total of 68 isoprenoid diether or tetraether lipids as well as two isoprenoid quinones (menaquinones) (Elling et al., 2014, 2016). 1G-GDGTs, 2G-GDGTs, 1G-OH-GDGTs, 2G-OH-GDGTs, archeol, methoxy-archeol, and HPH-GDGTs were the most abundant lipids of *N. maritimus*, which contained minor proportions of GTGT and monounsaturated GTGT detected using the normal phase liquid chromatography coupled to APCI-MS (Elling et al., 2014). These studies have further revealed that the changes of the membrane lipid composition can be a result of different growth rates, rather than pH or temperature, questioning the fundamental assumptions of a GDGT-based paleotemperature proxy, TEX_86_ (TetraEther indeX of 86 carbons). Particularly, the degree of GDGT cyclization, measured by the ring index, increases during later growth phases (Elling et al., 2014), at reduced oxygen concentrations (Qin et al., 2015), or lower ammonia oxidation rates (Hurley et al., 2016), either in batch or isothermal continuous culture experiments, suggesting that mesophilic *Thaumarchaeota* modulate their membrane composition to cope with bioenergetic stress, similar to other extremophilic *Crenarchaeota* and *Euryarchaeota* (Valentine, 2007).

With the use of LC-MS-based methodologies, the chemical diversity of the membrane lipids of *N. maritimus* (Elling et al., 2014, 2015, 2016), as well as its homeostatic membrane regulation to resist chronic energy stress (Qin et al., 2015; Hurley et al., 2016) have been revealed. However, much of the attention of the previous works was paid to a unique feature of the lipidome of *N. maritimus*, specifically, the cyclization in the GDGT membrane lipids since this could have been a prominent biosignature or palaeotemperature proxy observed in the sedimentary fossils in marine ecosystems (Schouten et al., 2002). However, the physiological responses to environmental stimuli, the membrane dynamics and structure, and the molecular interactions between membrane lipids and membrane-associated proteins are collective behavior of lipids, and are not restricted to the cyclization of GDGTs, or more accurately, incomplete saturation of the double bonds of the isoprenoid lipid precursors. These fundamental biomolecular mechanisms in archaea potentially manifest in the chemical and compositional diversity of their lipidome but have not been fully explored (Horai et al., 2019; Law and Zhang, 2019). While advances in chromatographic techniques and high-resolution mass spectrometers have contributed to the recent discoveries, deciphering the molecular puzzle of the lipidome necessitates further technological advances.

In recent years, ion-mobility spectrometry (IMS) technologies have emerged as a promising technique for lipid analysis (Kliman et al., 2011; Paglia et al., 2015b; Zheng et al., 2018; Harris et al., 2019; Tu et al., 2019) and could facilitate mass spectrometric analysis of the unique lipids from archaea. IMS is a gas- phase electrophoretic technique that separates ions based on their mobility in a chamber filled with a neutral buffer gas and subjected to an electrical field (Paglia et al., 2021). Because the mobility of an ion in the buffer gas depends not only on the ion’s mass and charge but also its shape and size, and the nature of the buffer gas, the technique has the potential to discriminate isomeric compounds that have the same mass-to-charge ratio but differ in their gas-phase geometries or conformations, as denoted by collision cross sections (CCSs) (Wu et al., 2020). CCS represents the rotationally averaged surface area of the ion which is available for interaction with the buffer gas (Pukala, 2019). Although the CCS value is not an intrinsic physicochemical property of the analyte ion but a quantity specific to the identity of the buffer gas, temperature, and electric field used for the measurement, it can be used as an additional molecular descriptor together with other orthogonal coordinates acquired by LC-MS to increase the specificity of compound identification (May et al., 2017).

While IMS can operate alone, it often interfaces with MS. Traveling wave ion mobility spectrometry mass spectrometry (TWIMS-MS) (Shah et al., 2013; Damen et al., 2014; Paglia et al., 2015a; Hankin et al., 2016; Hines et al., 2017; Sarbu et al., 2017; Hines and Xu, 2019; George et al., 2020; King et al., 2020) and drift-tube ion mobility spectrometry mass spectrometry (DTIMS-MS) (Kyle et al., 2016, 2018; Blaženović et al., 2018; Hinz et al., 2019; Leaptrot et al., 2019) systems are the most popular instrumental platforms for lipidomics. Nevertheless, there are still significant bottlenecks related to the resolving power of these IMS systems. These instrumental platforms have relatively low resolving power (*R* = 40–60), which is only sufficient for resolving ions differing in CCS values by ∼2-3%, including different classes of lipids, but not for most stereoisomers (ΔCCS < 1%) and enantiomers (ΔCCS ∼0.1%) (Masike et al., 2021). Recent technological advances in trapped ion mobility spectrometry (TIMS) (Vasilopoulou et al., 2020), structures for lossless ion manipulations (SLIM) (Wojcik et al., 2017), and cyclic ion mobility (cIM) systems (Colson et al., 2019) have shown promise for more demanding analysis. It has been demonstrated that at the expense of scanning rate and sensitivity, the resolving power of TIMS can be optimized up to ∼410, which is sufficient for the discrimination of the isomeric lipid species with ΔCCS < 1% (Jeanne Dit Fouque et al., 2019). Separation of isomeric phospholipids and glycolipids (gangliosides) have also been reported using the SLIM platform (Wojcik et al., 2017).

This work aimed to develop a novel analytical protocol using a popular TWIMS-MS platform for the analysis of isoprenoid lipids of marine archaea. It demonstrated a significant improvement upon an existing HPLC-Q-ToF-MS protocol that had already produced excellent results in the previous studies (Zhu R. et al., 2013; Elling et al., 2014, 2015, 2016). This novel approach allowed us to gain a deeper insight into the chemistry and diversity of archaeal lipids from *N. maritimus.* Gas-phase conformational properties of purified reference lipids encompassed the unique characteristics of archaeal isoprenoid lipids, such as methyl branching and cyclization, were investigated. The IM-MS measurement resulted in the first CCS library of archaeal lipids from *N. maritimus*. Our results demonstrated that the application of IM-MS was not only advantageous to lipidomics in biomedical science but potentially a viable approach to examining the lipidome of environmentally relevant microbes.

## Materials and Methods

### Study Design

**FIGURE 2:**
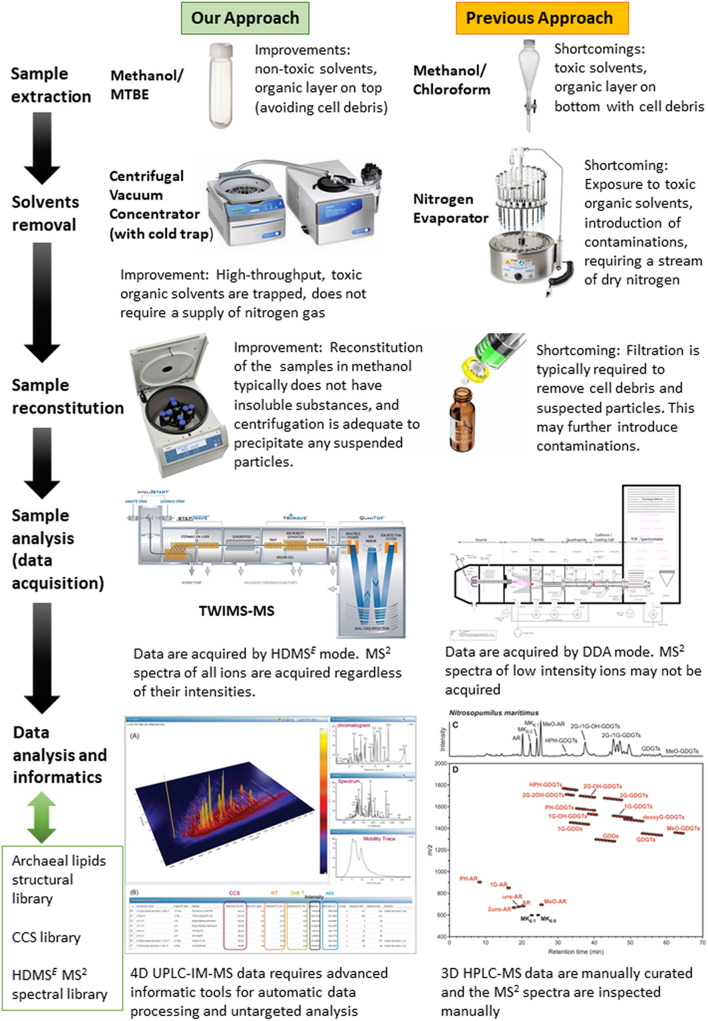
The enhancements brought by the UPLC-IM-MS protocol developed in this work in comparison to the protocol used in previous studies. The panel in the lower right corner is reproduced from Elling et al. (2016).

To evaluate the overall performance of this approach, the lipidome of a model marine group I *Thaumarchaeoton, N. maritimus* was characterized. The enhancements brought by our approach are summarized in Figure 2. For example, the lipidome of *N. maritimus* was extracted by methanol and methyl tert-butyl ether (MTBE) that substituted carcinogenic chloroform or dichloromethane used in the Bligh and Dyer protocol. The organic extracts containing the cellular lipids were characterized by UPLC-IM-MS. Unlike the protocol previously reported, data were acquired using a data-independent acquisition method (HDMS*^*E*^*) in both positive and negative ion modes, with methanol and ethanol (which is environmentally friendly) as the UPLC’s mobile phase. An archaeal lipids database was constructed for assisting spectral features annotation and is being made available to the community. Detailed procedures are described below.

### Reagents

Solvents and buffers for mass spectrometry analysis and lipid extraction were purchased from Merck (hyper grade methanol, gradient grade ethanol, LC grade MTBE, and LC-MS grade formic acid) or Fisher (optima grade acetonitrile, and 2-propanol). 1,2-diphytanoyl-*sn*-glycero-3-phosphocholine and 1,2-diarachidoyl-sn-glycero-3-phosphocholine were purchased from Avanti Polar Lipids (Alabama, United States), *beta*-L-gulopyranosyl-caldarchaetidyl-glycerol was purchased from Matreya, LLC (Pennsylvania, United States), and C46 GTGT was purchased from Pandion Laboratories LLC (Indiana, United States). Other reagents used for cell culturing and quantitative PCR assays have been described in our previous report (Law et al., 2021).

### Cell Culture, Treatment, and Lipid Extraction

*N. maritimus* strain SCM1 was cultivated in HEPES-buffered SCM (pH 7.5, 1 mM NH_4_Cl) as previously described in Könneke et al. (2005) and Martens-Habbena et al. (2009) with an addition of 1 mg/ml catalase solution. Cell growth was monitored by nitrite production, and the purity of the culture was monitored by quantitative real-time PCR (qPCR) analysis (Yu et al., 2005). Further details have been given in Law et al. (2021) previously. Cells were harvested by filtering through a 0.22 μm Polyvinylidene Fluoride (PVDF) filter (Whatman) after reaching the stationary phase. Filters were stored at –80°C until extraction. An MTBE-based lipid extraction procedure is used in this study as it has superseded the chloroform-based approach commonly used (Willers et al., 2015). MTBE is less toxic than chloroform. It is less dense than water and the organic phase retaining the lipids sits on the upper layer while hydrophilic compounds and salts are enriched in the lower aqueous phase. This allows the collection of the lipid-containing phase without contacting either the aqueous phase or non-extractable residues at the bottom of the extraction tube, and thus affording a contamination-free collection of the two main liquid phases (Salem et al., 2016). The extraction efficiency is comparable to the Bligh and Dyer protocol (Züllig et al., 2020). A recent study, comparing MTBE and chloroform-based protocols, suggests that the MTBE method is more efficient for glycerophospholipids, ceramides, and unsaturated fatty acids, while the chloroform protocol is superior for saturated fatty acids and plasmalogens (López-Bascón et al., 2018), and thus meeting the challenges and criteria of this work. One caveat of the MTBE method is that MTBE is more polar than chloroform. As a result, a carry-over of water is inevitable. It is recommended to use a centrifugal concentrator to reduce the sample to dryness.

Lipids were extracted at room temperature as previously described (Matyash et al., 2008). In the first step, biomass captured on a PVDF membrane was extracted by 4 ml of MTBE and 1.2 ml methanol (10:3; *v/v*) in a clean Teflon tube (Thermo Scientific Nalgene Oak Ridge high-speed centrifuge tubes). The tube was placed on a tube revolver (Thermo Fisher Scientific) and rotated for an hour. Thereafter, 1 ml of water was added to the extract to induce phase separation. The extract was vortexed and centrifuged to allow the partition of polar metabolites and lipids into the aqueous and organic phases. The upper organic phase was collected in a clean Teflon tube, whereas the lower aqueous phase and the biomass were re-extracted two additional times. The combined organic fraction was reduced to dryness using a centrifugal concentrator (Christ RVS 2-18 CD plus, Osterode am Harz, Germany) and the dried samples were stored at –30°C until analysis. The aqueous fraction was discarded.

### Liquid Chromatographic Ion Mobility Mass Spectrometry Analysis

A reverse-phase liquid chromatographic method was adopted from Zhu C. et al. (2013) with chromatographic gradient modified to reduce analytical time. An analytical ACE Excel 2 SuperC18 column from Advanced Chromatography Technologies Ltd., Aberdeen, Scotland was used. It had been demonstrated that this chromatographic method has higher performance for archaeal ether lipid analysis relative to a normal-phase chromatographic method based on a diol column (Zhu C. et al., 2013). The SuperC18 column was also found to perform better (lower back pressure) than a similar CORTECS C18 column from Waters despite having a slightly larger particle size. As a result, an elevated column temperature was not required and the chromatographic resolution was maintained by minimizing longitudinal diffusion.

Methanol and ethanol were used as the eluents to minimize interferences. This modification was essential as the use of methanol-isopropanol mobile phase and aqueous ammonia modifier as previously reported was found to extravagate background interferences under ion mobility mode, especially in the mass region 1,200–1,800 *m/z*. The reason for this was not known although these interferences were a lot minor in ToF mode when IMS was switched off. Moreover, an ion mobility enhanced data-independent acquisition (DIA) method (HDMS*^*E*^*) was chosen over data-dependent acquisition (DDA) used in the previous studies, as HDMS*^*E*^* offered several advantages over DDA, including the acquisition of fragmentation spectra indiscriminately with the peak’s intensity. The spectral quality of HDMS*^*E*^* spectra should be comparable with MS^2^ obtained by DDA.

Chromatographic separation was performed using an AQUITY UPLC system (Waters, Manchester, United Kingdom) equipped with an ACE Excel 2 SuperC18 column (2 μm, 2.1 × 150 mm). Solvent A was methanol and solvent B was ethanol. Both solvents were modified with 0.1% aqueous ammonia and 0.04% formic acid. The strong wash solvent was 2-propanol. The linear gradient started at 100% A, held for 4 min, then increased to 50% B at 10 min, and further increased to 99% B at 30 min and held for another 4 min. The gradient returned to 100% B at 34.1 min and re-equilibrated for 2 min. The flow rate was 0.40 mL/min. The column temperature was maintained at 45°C. The sample manager was maintained at 7°C.

Mass spectrometry analysis was conducted on a Waters Synapt G2-S*i* (Waters, Manchester, United Kingdom) equipped with an electrospray ionization (ESI) source. Data acquisition was performed with HDMS with extended dynamic range or HDMS*^*E*^* operated in resolution mode (resolving power > 30,000). The mass analyzer was mass calibrated with 2 μg/μL sodium iodide solution. The ion mobility was calibrated with Waters Major Mix IMS/Tof Calibration Kit according to the vendor’s instructions. One ng/μL leucine-enkephaline was used as the lockspray solution. The system was controlled via MassLynx software, version 4.2 SCN 983.

Lipid extracts were analyzed with the following parameters: The capillary voltage was 2.8 and 2.2 kV in positive and negative ion mode, respectively. The sample cone was 40 V, source temperature was 120°C. Cone gas was 50 L/h, desolvatization gas was 600 L/h and nebulizer gas was 6.5 Bar. Trap DC bias was 60 V, Trap DE exit was 3 V. IMS wave velocity was 500 m/s, wave height was 40 V, transfer wave velocity was 179 m/s, and wave height was 4 V. Samples were reconstituted in 150 μl of methanol. Hundred microliter was transferred to a sample vial, the remaining was pooled to prepare a QC sample. Ten microliter of the sample was injected into the system. Data were acquired in a continuum from 50 to 2,000 Da, from 3.5 to 34 min. The transfer collision energy was ramped from 40 to 120 V. Scan time was 0.4 s. Solvent blanks, extraction blanks, and QCs were analyzed at the same time.

### Construction of Archaeal Lipid Structure Database

An archaeal lipids database was constructed with Progenesis SDF Studio ver. 1.0. A brief introduction was given in our previous work (Law and Zhang, 2019). The library contained a total of 953 structures, including 26 lipids from LipidMaps, 162 lipids from LipidBank, 121 lipids from PubChem, 24 lipids and lipid intermediates from MetaCyc, 64 lipids and metabolites from ChEBI, 32 carotenoids from ChemSpider and Carotenoids database, and 524 lipids were from literature or speculated from literature data. Redundancy (repeated entries of the same lipid from database sources) was kept. The SDF library was subsequently used to identify compounds by Progenesis QI.

### Data Processing and Analysis

UNIFI software (Waters, Manchester, United Kingdom) ver. 1.9 SR4, was used to visualize the raw IM-MS spectra (Rosnack et al., 2016). The raw spectra were further processed by Progenesis QI (Non-linear Dynamics, Newcastle upon Tyne, United Kingdom) ver. 2.4, and underwent automatic deconvolution and alignment. Peak picking was set to default sensitivity with a minimum peak width of 1.5 s. Data were normalized by the default method of the software, normalize to all compounds.

Spectral features were annotated against an in-house Archaeal Lipid Library (see the section above), LIPID MAP database (release 20201001) (Schmelzer et al., 2007), and BioCyc *N. maritimus* SCM1 library (ver. 24.0), using the Progenesis MetaScope algorithm (version 1.0.6901.37313), with precursor and theoretical fragmentation tolerance with a relative mass error of 5 ppm. Spectral characteristics, including mass errors, isotope similarities, and similarities between experimental and *in silico* fragmentation spectra, were used for accessing the confidence of the assignment, and a quantitative scoring system was used to estimate the confidence of the metabolite assignments (Creek et al., 2014). The software calculated the similarity of each spectral characteristic and summed them to an overall confidence score (maximum score 60). Annotations of the spectral features were tentatively assigned from lipid candidates with an overall score ≥ 47. Annotations of the spectral features were therefore level 2b (probable structures, with a unique lipid matched to the spectral feature), or level 3 (tentative candidates, with two or more isomeric lipids matched to the spectral feature and were indistinguishable by MS^2^ or IMS) based on the revised reporting standards proposed by Metabolomics Standards Initiative (Schymanski et al., 2014).

## Results and Discussion

### IM-MS Characterization of Representative Archaeal Lipids

One of the main features distinguishing archaeal lipids from bacterial and eukaryotic lipids is their hydrocarbon chains, which are characterized by a repetition of a five-carbon unit with a methyl group at every fourth carbon of a saturated isoprene unit (Caforio and Driessen, 2017). These branched, saturated structures are thought to enhance their chemical resistance to hydrolysis and oxidation and permit archaea to withstand extreme environmental conditions. However, how does this unique feature modify the chemistry of the lipid molecules and so regulate the collective behavior of the membrane?

**FIGURE 3:**
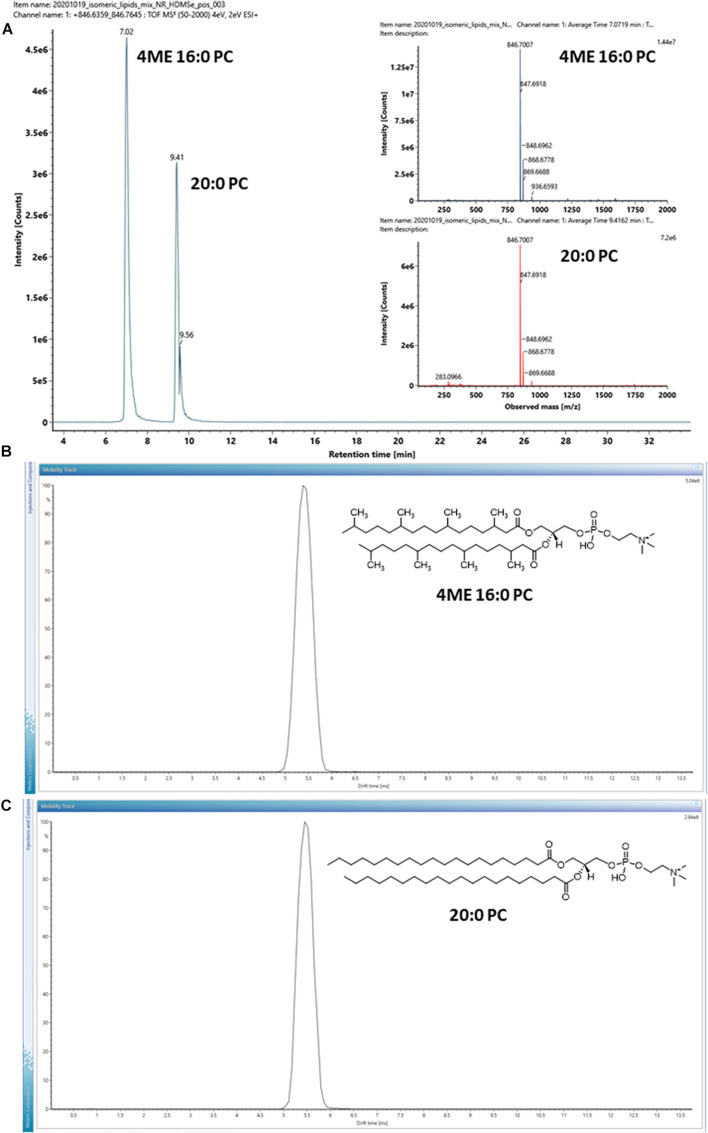
**(A)** EIC of a pair of isomeric lipids, 1,2-diphytanoyl-*sn*-glycero-3-phosphocholine (4ME 16:0 PC) and 1,2-diarachidoyl-*sn*-glycero-3-phosphocholine (20:0 PC) analyzed under positive ion mode. Inserts are MS^1^ mass spectra of the isomeric lipid pair. **(B,C)** Arrival time distributions of the protonated form [M + H]^+^ of the isomeric pair. Inserts show their chemical structures. The arrival time distributions are almost identical, despite the differences in their hydrocarbon tails.

A pair of isomeric diester lipids, 1,2-diphytanoyl-*sn*-glycero-3-phosphocholine (4ME 16:0 PC) and 1,2-diarachidoyl-*sn*-glycero-3-phosphocholine (20:0 PC), were chosen and analyzed by LC-IM-MS. Their structures are shown in the inserts of Figure 3. 20:0 PC was chosen to represent a typical linear eukaryotic lipid, whereas 4ME 16:0 PC was chosen to represent a typical branched isoprenoid diether lipid of archaea. It should be stressed that while the isomeric pair was chosen because of their hydrocarbon chains, the bond angle of ether and ester linkages are not identical, ether lipids are slightly larger than ester lipids with the same mass^1^.

The isomeric lipids were effectively separated by chromatography with 20:0 PC stronger retained by the C18 chromatographic substrate (Figure 3A). On the other hand, the HDMS*^*E*^* MS^2^ spectra of the isomer pair were almost identical (Supplementary Figure 1). Distinguishing the isomeric lipids had to rely on their chromatographic characteristics and hence the availability of reference compounds analyzed under the same experimental conditions.

The ion mobility characteristics of the isomeric pair were further measured by IMS and the arrival time distributions of the protonated form [M + H]^+^ of the ions are shown in Figures 3B,C. The drift-times measured by IMS had only a minor difference with < 0.1 ms under the instrumental parameters used, although the isomeric lipids might still be discriminated by the CCS values of the adducts or dimers (Table 1). For example, the CCS values of the [2M + Na]^+^ ion of the isomeric lipids differed by observable 6 Å^2^.

**TABLE 1:** The CCS values of various adducts of the isomeric lipid pair.

**Compound**	**Retention time (min)**	**Adduct**	**Experimental CCS (Å^2^)**	Theoretical CCS (Å^2^)*
1,2-Diphytanoyl-*sn*-glycero-3-phosphocholine	7.02	M + H	317.46	303.7
		M + K	332.96	311.3
		2M + H	473.90	
		2M + Na	476.33	
1,2-Diarachidoyl-*sn*-glycero-3-phosphocholine	9.38	M + H	317.46	308.8
		M + Na	320.88	308.1
		M + K	331.24	314.9
		2M + H	471.40	
		2M + Na	470.10	

*Theoretical CCS values were predicted by CCSbase v1.1 using isomeric SMILES codes from the PubChem database.

These results suggested that, in contrast to the straight-chain diester lipids with the same number of carbons (20:0 PC), methyl branching of the diester lipid (4ME 16: PC) reduces lateral interactions with other lipid-like molecules in the membrane, as they would have with the C18 chromatographic substrate, thereby increasing the fluidity of lipid bilayers. However, as measured by their ion mobility, the overall size of the gas-phase ions is not significantly affected by methyl branching. This observation offers an insight into the adaptability of the archaeal membrane through the structural diversity of lipids. If the isoprenoid chain of diether lipids of archaea are chemically similar to the branched diester counterparts, the biosynthesis of both diphytanylglycerol diether and dibiphytanylglycerol tetraether lipids (which are the end-to-end coupling of two dibiphytanylglycerol diether molecules) in archaea, allow them to have a wider range of instruments to accommodate the environmental changes by alternating the stiffness of the membrane without significantly alternating the thickness of the membrane.

**FIGURE 4:**
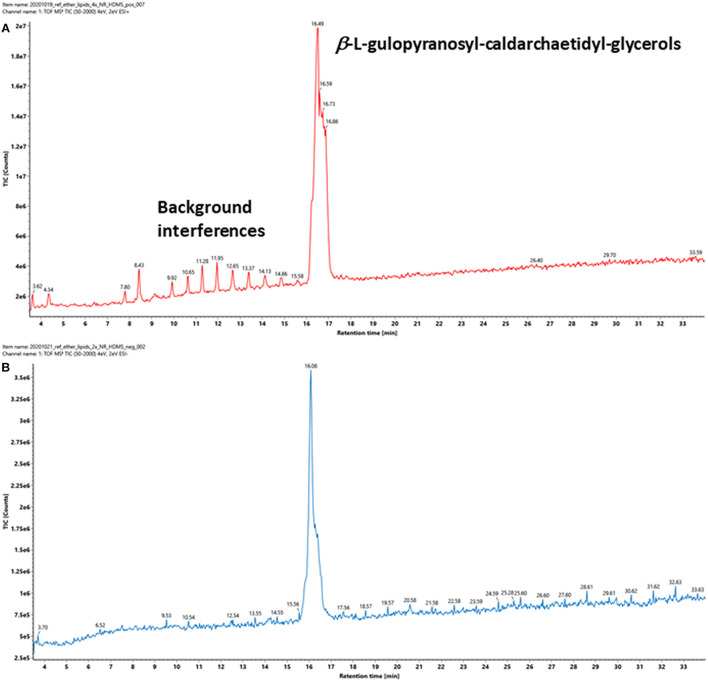
TIC of a commercially available tetraether monosaccharide phospholipid standard acquired under **(A)** positive and **(B)** negative ion modes. The information provided by the vendor describes it contains a β-L-gulopyranosyl-caldarchaetidyl-glycerol, a glycerophosphate, and a gulosylpyranoside monosaccharide linked via a tetraether chain. The phospholipid is synthesized by *Thermoplasma acidophilum*, with purity > 95%.

A representative archaeal phosphoglycerol GDGT lipid, β-L-gulopyranosyl-caldarchaetidyl-glycerol, obtained from a commercial supplier, was initially chosen to optimize the IMS parameters. The vendor described the product as a tetraether monosaccharide phospholipid of high purity (>95%) and is the main phospholipid of thermoacidophilic archaeon, *Thermoplasma acidophilum*. It contains a glycerophosphate and a gulosyl pyranoside monosaccharide linked via a tetraether chain. Subsequent analysis revealed that the product contained a mixture of β-L-gulopyranosyl-caldarchaetidyl-glycerols with cyclopentane rings from 0 to 4, which were poorly separated chromatographically, resulting in a relatively broad chromatographic peak (Figure 4).

**FIGURE 5:**
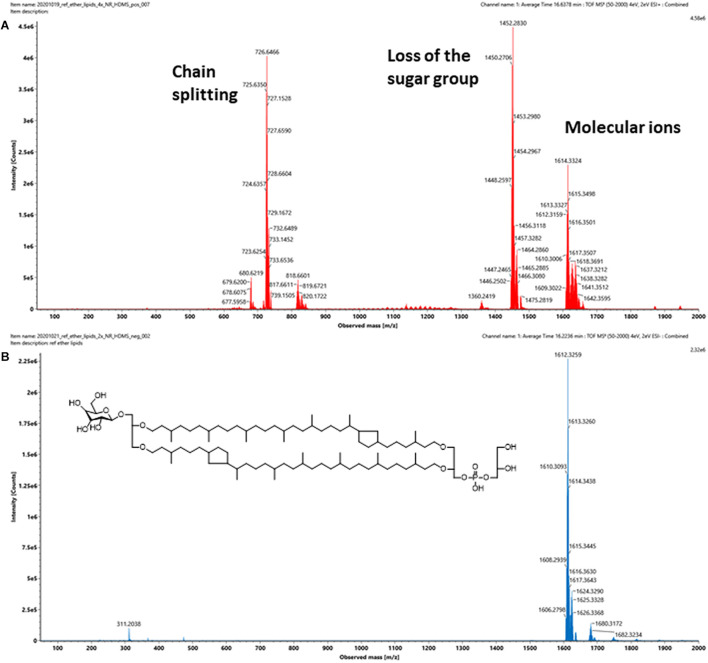
**(A)** Positive and **(B)** negative ion mass spectra of a commercially available intact polar GDGT reference standard that contains a mixture of at least five β-L-gulopyranosyl-caldarchaetidyl-glycerols. Insert shows the chemical structure of the major component, which contains two cyclopentane rings. The number of spectral features detected is typically a lot higher than the number of lipids in a sample. The challenges associated with the formation artifacts could also be avoided or circumvented in negative mode, as uncomplicated mass spectra of intact GDGTs could be obtained.

Intact GDGT lipids from archaeal cellular extracts are typically analyzed using ESI in positive ionization mode. However, background interferences are frequently encountered and obscure the analysis (Figure 4A). These signals were typically synthetic polymers and could not be completely avoided. In addition to formation and various adducts, intact GDGT lipids fragment extensively in positive mode, leading to many degenerated features and artifacts (Figure 5A). As a result, multiple signals derived from the same lipid are observed in the mass spectra, complicating automatic chromatographic deconvolution and spectral annotation. Moreover, these artifacts can cause other problems, especially when the fragment ions correspond to isomeric species of other endogenous lipid species in the samples and lead to incorrect annotation and/or false identification (Hu et al., 2020). Owing to the presence of a phosphate group, an option to mitigate these problems was to analyze β-L-gulopyranosyl-caldarchaetidyl-glycerols in negative ionization mode and to avoid the complications associated with background interferences and in-source fragmentation (Figures 4B, 5B). Furthermore, with the addition of the negative data, annotated spectral features could be grouped manually to circumvent the problems associated with the false identification of fragment ions in the positive analysis (see the subsequent analyses of the cell cultures).

**FIGURE 6:**
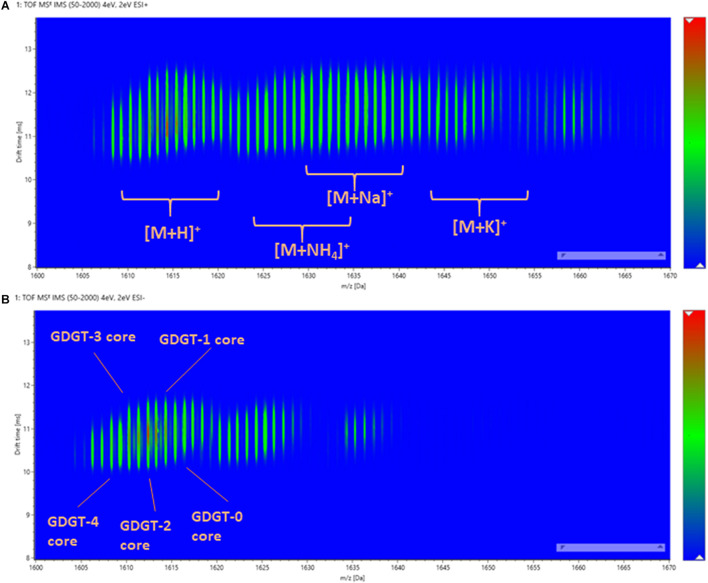
The drift-time distribution of β-L-gulopyranosyl-caldarchaetidyl-glycerols measured in **(A)** positive and **(B)** negative ionization modes.

In contrast to methyl branching, the cyclopentane rings of GDGTs have a much greater influence on the overall size of the molecule measured by ion mobility. The ion mobility characteristics of β-L-gulopyranosyl-caldarchaetidyl-glycerols were qualified under both positive and negative modes (Figures 6A,B). Due to incomplete chromatographic separation, a variety of adduct and isotopic peaks was observed in the drift-time distribution plot. However, the lipids with a different number of cyclopentane rings could still be distinguished by their *m/z*. A noticeable feature was that the drift-time distributions of polar GDGTs were much broader than typical ester lipids (e.g., in contrast to 20:0 PC and 4ME 16:0 PC). This suggested that the C40 isoprenoid chains of GDGTs are relatively elastic and multiple gas-phase ion conformations exist. This was in contrast to the conventional view that archaeal tetraether lipids have higher chemical stability than diester lipids because of a reduced segmental motion of tertiary carbon atoms (Albers et al., 2006). The chemical stability of archaeal membrane is likely owing to the collective thermodynamic behavior of isoprenoid ether lipid molecules in a liquid-crystalline state, rather than a specific chemical property of the isoprenoid ether molecules (Chugunov et al., 2014; Pineda De Castro et al., 2016b,a; Vitkova et al., 2020).

**FIGURE 7:**
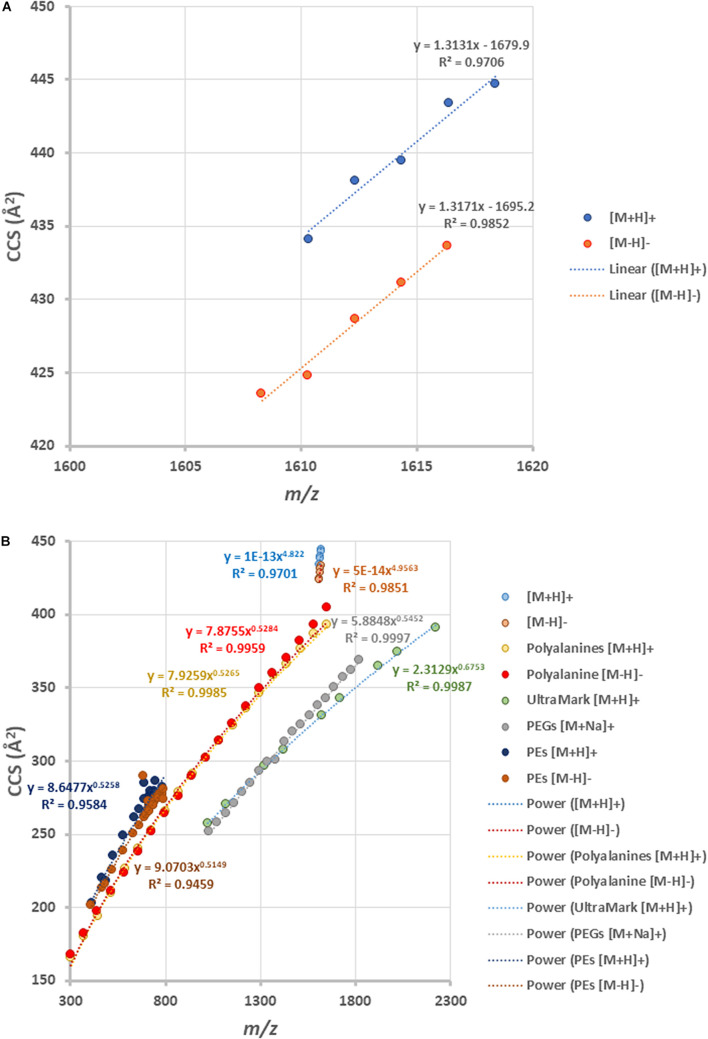
**(A)** Ion mobility-mass correlation trendlines of β-L-gulopyranosyl-caldarchaetidyl-glycerols [M + H]^+^ and [M-H]^–^ ions. The trendlines are linear fitted. **(B)** Comparing the ion mobility-mass correlation trendlines of β-L-gulopyranosyl-caldarchaetidyl-glycerols with that of glycerophosphoethanolamines (PEs) [M + H]^+^ and [M-H]^–^ ion, polyalanines [M + H]^+^ and [M-H]^–^ ions, Ultramark [M + H]^+^ ions and PEGs [M + Na]^+^ ions. The trendlines are power fitted.

By plotting the CCS values of each ion peak against the *m/z* values of β-L-gulopyranosyl-caldarchaetidyl-glycerols with core GDGT-4 to GDGT-0, a linear correlation trendline was obtained for both [M + H]^+^ and [M-H]^–^ ions (Figure 7A). When the gradients of the trendlines of β-L-gulopyranosyl-caldarchaetidyl-glycerols were compared to other structural series of molecules, such as polyalanines, polyethylene glycols (PEGs), and Ultramark (fluorinated phosphazenes), and glycerophosphoethanolamines (PEs), the trendlines of β-L-gulopyranosyl-caldarchaetidyl-glycerols were much steeper than these classes of compounds (Figure 7B). CCSs intrinsically correlate with molecular masses. This characteristic leads to typically power-fitted regression trendlines obtained with IM-MS analysis of a class of compounds. But factors other than mass or chemical class can also affect how a molecule is packed in three-dimensional space. Our observations reinforce the proposition that the addition of each cyclopentane ring in GDGTs effectively reduces their molecular size and potentially the membrane thickness of archaeal cells (Gabriel and Chong, 2000; Chong et al., 2012). Taking together, having the instruments to regulate the stiffness and the thickness of the plasma membrane through the collective behavior of individual isoprenoid ether lipid molecules in the membrane might explain the success of archaea to adapt to a wide range of environments.

### UPLC-IM-MS Analyses of the Methyl Tert-Butyl Ether Cellular Extracts of *N. maritimus*

**FIGURE 8:**
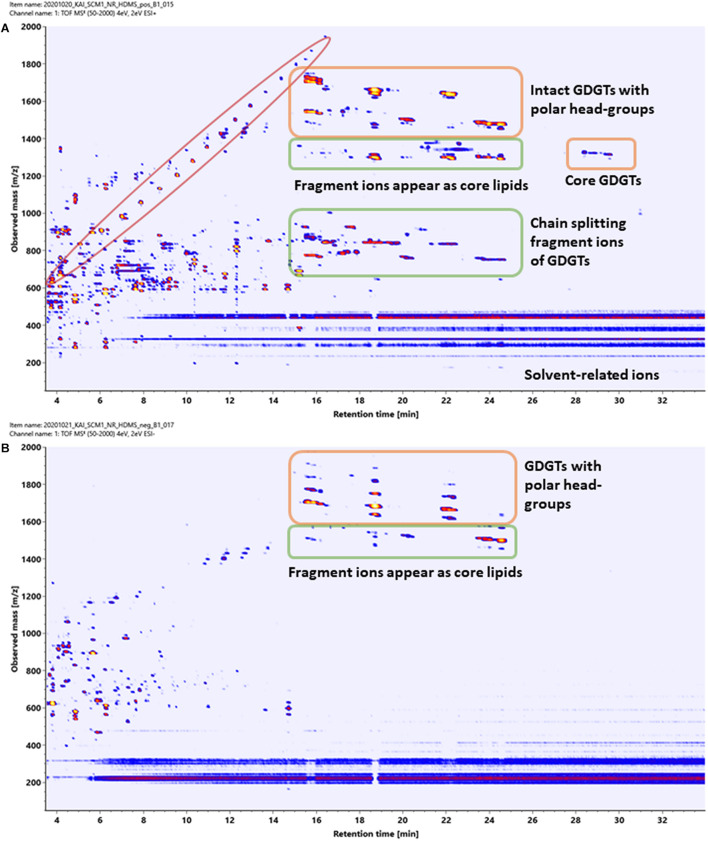
Density maps (*mz*-rt plots) of an experimental cellular lipid extract acquired in **(A)** positive and **(B)** negative ionization modes. In positive mode, in-source fragmentation of intact polar GDGT lipids generated core GDGT lipids as well as other fragment ions. Care should be taken to avoid misannotation. In negative mode, only adduct ions of intact polar GDGT lipids were observed. Polymer ions formed a diagonal group whereas solvent-related background ions appeared as horizontal bands.

Two-dimensional density maps were often used in literature to illustrate the number of lipids detected by HPLC-Q-ToF-MS analyses (Becker et al., 2013, 2016), although many of the spots in the density maps reported were not annotated. Figures 8A,B show the density maps of a cellular lipid extract of *N. maritimus* acquired in positive and negative modes, respectively. There were hundreds of unique spots in the density maps of the cellular lipid extracts even after considering the background ions observed in the extraction blanks (Supplementary Figure 3). We wondered that does the complexity of these maps provides a visual estimation of the diversity of the lipidome of *N. maritimus*? If so, could there be more archaeal lipids in the natural or biological systems than that have ever been analyzed by low-resolution mass spectrometric methods?

Estimating the number of the spots in the density maps of the extract of *N. maritimus* implied that there were several hundred or more lipid species in the lipidome of *N. maritimus.* Nevertheless, as shown by the IM-MS analyses of representative GDGT standard in the previous section, many ions observed in the positive spectra were in-source fragment ions, adducts, and artifacts. Observing the number of spots in the density maps would overestimate the diversity of lipids in the lipidome. Furthermore, a small proportion of lipids might have come from bacterial contaminations or were introduced during cultivation or sample preparation. The data acquired in negative mode, however, did not provide a more accurate estimation of the lipid diversity, as most archaeal lipids do not have phosphate or sulfate groups or were not ionized in negative ionization mode. Core lipids for example could only be detected in the positive analyses.

To better answer this question, the dataset acquired under both positive and negative modes was further processed by Progenesis QI. After data pre-processing and statistically removing the background ions detected in the extraction blanks, the deconvoluted spectral features were annotated to lipids by accurate mass, isotope similarity, and HDMS*^*E*^* spectra against the lipids in an in-house library and two other public databases. Their HDMS*^*E*^* spectra were matched against the metfrag *in silico* fragmentation. Approximately 90 spectral features were annotated with lipids (Supplementary Table 1), including lipids possibly of bacterial origins (highlighted in yellow in Supplementary Table 1). Adducts of archaeal lipids other than typically reported [M + H]^+^, [M + Na]^+^, and [M + NH_4_]^+^ were also detected. Our observations were mostly consistent with those reported previously (Elling et al., 2014, 2016). However, because our approach did not utilize manual curation (manually generating extracted ion chromatograms and accessing the identity of the features), but to qualify the confidence of the annotation by assessing three major spectral characteristics, low abundant lipids that did not produce high-quality MS^2^ spectra or projected isotope distribution might not have been accepted for identification, as their overall confidence score was lower than the cut-off score 47.

Generally, lipids are separated mainly by their alkyl chain hydrophobicity by reverse-phase chromatography. As a result, lipids with the same headgroup and only slight differences in the core lipid structure can be effectively separated (Wörmer et al., 2013, 2017). However, as most archaeols or GDGTs are composed invariably of C20 or C40 isoprenoid chains, the hydrophilic polar headgroups do play a significant factor in reverse-phase chromatographic separation. The overall elution order of archaeal diether and tetraether lipids was dominated by the isoprenoid chains’ length, with a variety of archaeols eluted first followed by an assorted of GDGTs (Supplementary Table 1). Within the group of diether or tetraether lipids, the lipids with more hydrophilic headgroups or hydroxyl modifications were eluted first, while the core lipids eluted last. Intact polar tetraether GDGT lipids that differed only by a cyclopentane ring were largely co-eluted, while core GDGT lipids were mostly separated, though their peak intensities were relatively low. Hence, the overall increase in hydrophobicity owing to cyclopentane rings was inconspicuous in the presence of other functional groups. The CCS values of the detected lipids are also shown in Supplementary Table 1. To the best of our knowledge, it was the first experimental measured CCS values of archaeal lipids reported and could be used in the future IM-MS analysis of archaeal lipids.

A pair of isomeric diglycosyl-dihydroxyl-GDGT-2 lipids was detected (highlighted in green in Supplementary Table 1). The isomeric pair was separated by chromatography by 2 min and were thought to be glycosylated with two hexose moieties at both ends (eluted earlier), and glycosylated with one dihexose moiety at one of the glycerol moieties (eluted later) (Besseling et al., 2018). This resulted in a small reduction of the CCS values measured by IMS. This was consistent with an observation reported in a previous study (Elling et al., 2014) that a sequence of compounds with identical molecular mass and MS^2^ spectra as 2G-GDGTs were detected, but these 2G-GDGTs were separated by their chromatography conditions by 4 min.

**FIGURE 9:**
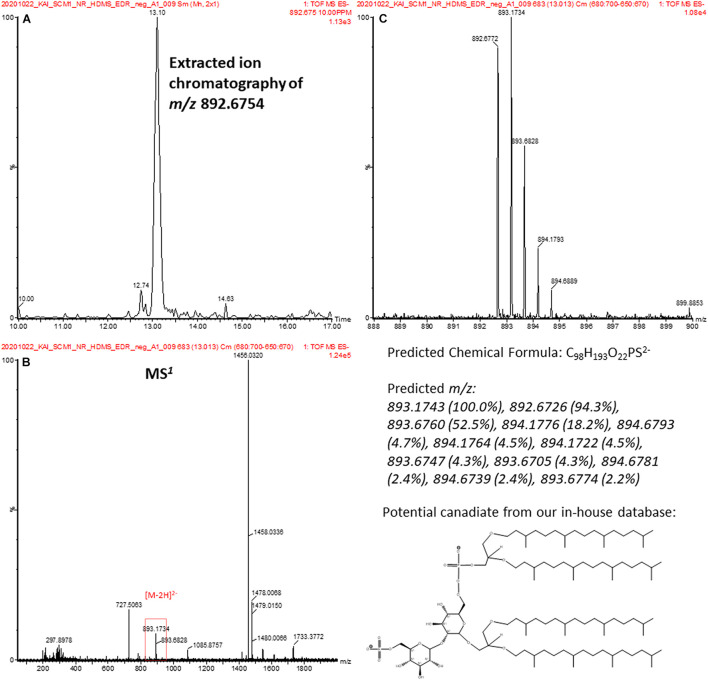
**(A)** EIC confirms a detection of a spectral feature at 13.1 min. **(B)** The MS^1^ spectrum of the spectral feature in **(A)**. **(C)** The isotopic pattern of the doubly charged ion at *m*/*z* 892.2754 in **(B)** and is consistent with the predicted pattern of a candidate lipid, 2-(6′-sulfo-β-D-mannosyl)-6-archaetidyl-α-D-glucosylarchaeol, in our database.

Characterization of archaeal lipids has mostly been performed in positive mode. Phosphate-containing and other negatively charged lipids have rarely been reported (de Souza et al., 2009). One of the features was annotated to a sulfate- and phosphate-containing archeol in negative analysis (highlighted in red in Supplementary Table 1). The spectral data were accessed manually. An extracted ion chromatography confirmed the detection of the feature at *m/z* 892.6754 (Figure 9A). The feature corresponded to a doubly charged [M-2H]^2–^ ion (Figure 9B) with a unique isotopic pattern (Figure 9C). The isotopic pattern suggested that the chemical formula of the molecular ion could probably be C_9__8_H_19__5_O_2__2_PS^2–^, and this matched to 2-(6′-sulfo-β-D-mannosyl)-6-archaetidyl-α-D-glucosylarchaeol in our in-house library. Unfortunately, a reliable MS^2^ spectrum was not generated because of the signal intensity. To ascertain the putative assignment, an authentic reference standard would be required for verification.

### Comparison to Manual Curation

Comprehensive characterization of the lipidome with high-resolution mass spectrometry analysis has conventionally been performed with manual curation (Fauland et al., 2011). This method has the advantage to reveal low abundant lipid species that do not generate reliable MS^2^ spectra. These low abundance species may also be filtered off by automatic data pre-processing and are not seen in the outputs.

In manual curation, accurate mass and accurate retention are important criteria for identification. The method of choice is reversed-phase liquid chromatography, where the separation of lipid species within one lipid class is mainly based on the interaction between the hydrophobic stationary phase and hydrocarbon chains of the lipid. Identification of lipid species is possible by retention time even in the absence of reliable MS^2^ spectra provided that any other species of the series has a reliable MS^2^ spectrum (Fauland et al., 2011).

**FIGURE 10:**
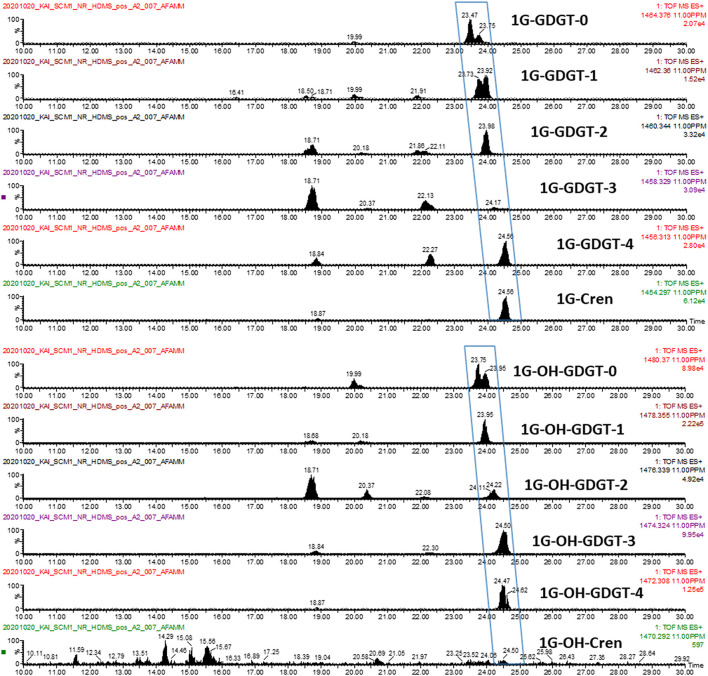
Manual curation: EICs of 1G-GDGTs (top) and 1G-OH-GDGTs (bottom) [M + H]^+^ ions.

A series of glycosidic GDGTs were chosen as examples since they were among the most abundant lipid species in the lipidome of *N. maritimus* and their accurate masses were listed in Table 2. Figure 10 shows the chromatograms of monoglycosyl-GDGTs **(top)** and monoglycosyl-hydroxyl-GDGTs **(bottom)** [M + H]^+^ ions. Despite the interferences, it was clear that GDGTs eluted at around 24 min. An incremental increase in retention time was observed with the number of cyclopentane rings. An addition of hydroxyl moiety reduced the hydrophobicity of GDGTs and so the chromatographic retention.

**TABLE 2:** Manual curation using an accurate mass and retention approach.

**Lipids**	**[M + H]^+^**		**[M + NH_4_]^+^**		**[M + Na]^+^**		**Manual**	**Progenesis**
1G-GDGT-0	1464.3755	✓	1481.4020	✓	1486.3574	✓	✓	✓
1G-GDGT-1	1462.3598	✓	1479.3864	✓	1484.3418	✓	✓	×
1G-GDGT-2	1460.3442	✓	1477.3707	✓	1482.3261	✓	✓	×
1G-GDGT-3	1458.3285	×	1475.3551	✓	1480.3105	✓	✓	✓
1G-GDGT-4	1456.3129	✓	1473.3394	✓	1478.2948	✓	✓	×
1G-Cren	1454.2972	✓	1471.3238	✓	1476.2792	✓	✓	×
1G-OH-GDGT-0	1480.3704	✓	1497.3970	✓	1502.3524	✓	✓	✓
1G-OH-GDGT-1	1478.3548	✓	1495.3813	✓	1500.3367	✓	✓	✓
1G-OH-GDGT-2	1476.3391	✓	1493.3657	✓	1498.3211	✓	✓	✓
1G-OH-GDGT-3	1474.3235	✓	1491.3500	✓	1496.3054	✓	✓	×
1G-OH-GDGT-4	1472.3078	✓	1489.3344	✓	1494.2898	✓	✓	×
1G-OH-Cren	1470.2922	×	1487.3187	✓	1492.2741	✓	✓	×
2G-GDGT-0	1626.4283	✓	1643.4549	✓	1648.4103	✓	✓	×
2G-GDGT-1	1624.4127	✓	1641.4392	✓	1646.3946	✓	✓	×
2G-GDGT-2	1622.3970	✓	1639.4236	✓	1644.3790	✓	✓	✓
2G-GDGT-3	1620.3814	✓	1637.4079	✓	1642.3633	✓	✓	✓
2G-GDGT-4	1618.3657	✓	1635.3923	✓	1640.3477	✓	✓	✓
2G-Cren	1616.3501	✓	1633.3766	✓	1638.3320	✓	✓	×
2G-OH-GDGT-0	1642.4232	✓	1659.4498	✓	1664.4052	✓	✓	✓
2G-OH-GDGT-1	1640.4076	✓	1657.4341	✓	1662.3895	✓	✓	×
2G-OH-GDGT-2	1638.3919	✓	1655.4185	✓	1660.3739	✓	✓	✓
2G-OH-GDGT-3	1636.3763	✓	1653.4028	✓	1658.3582	✓	✓	✓
2G-OH-GDGT-4	1634.3606	✓	1651.3872	✓	1656.3426	✓	✓	×
2G-OH-Cren	1632.3450	×	1649.3715	×	1654.3269	✓	×	×

*A GDGT lipid is considered “detected” if the chromatographic peaks of two or more of its adducts are observed and the elution order of the chromatographic peaks agrees with the relative hydrophobicity of the lipids.*

One data curation approach sums the scans of [M + H]^+^, [M + NH_4_]^+^, and [M + Na]^+^ to increase the signal-to-noise ratio of the extracted ion peaks (Zhu C. et al., 2013). An alternative approach inspects the ion chromatograms of each of these adducts individually to increase the confidence of identification. We chose the latter method to minimize the likelihood of false identification, and the chromatograms of [M + NH_4_]^+^ and [M + Na]^+^ adducts were evaluated separately.

While the chromatograms of monoglycosyl-GDGTs [M + NH_4_]^+^ and [M + Na]^+^ adducts were similar to that of [M + H]^+^ (Supplementary Figures 3, 4, top), the chromatographic peaks of monoglycosyl-hydroxyl-GDGTs [M + NH_4_]^+^ adducts were divided into two sets (Supplementary Figure 3, bottom) and were labeled as set 1 and set 2 in the figure. Only set 2 was observed in the chromatograms of [M + Na]^+^ adduct (Supplementary Figure 4, bottom). A similar observation was not reported previously and the reason for this was unknown. Hence, manual curation was not free from ambiguity.

**FIGURE 11:**
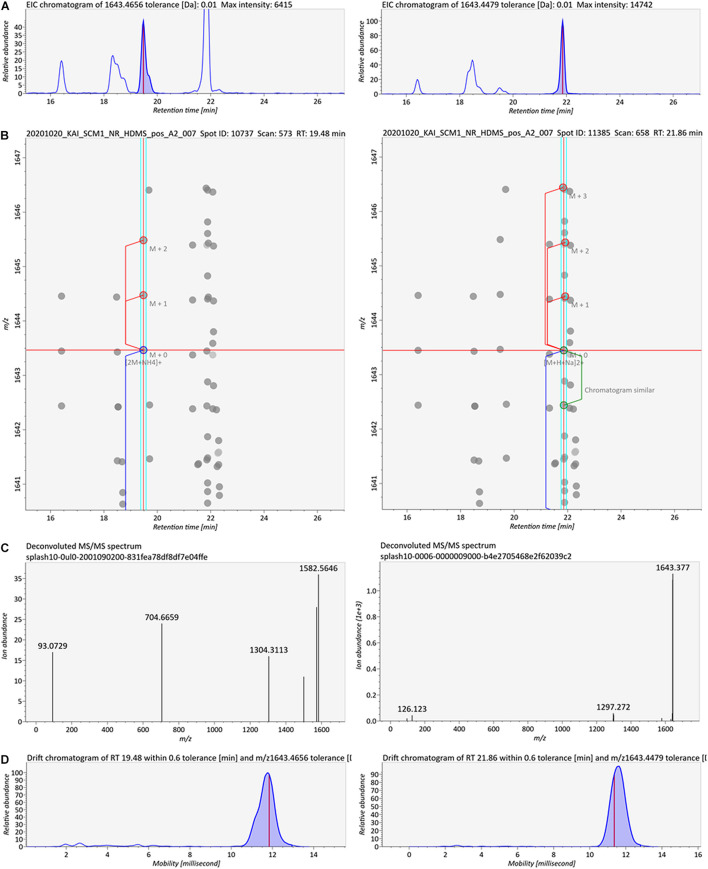
Spectral characteristics of the 2G-GDGT-0 type I and II structural isomers ([M + NH_4_]^+^). **(A)** EICs of the structural isomers that were eluted 2 min apart, **(B)** deconvolution of the spectral features and isotopic ions, **(C)** deconvoluted MS^2^ spectra of the isomers, and **(D)** ion mobility spectra of the isomers.

Two sets of peaks were observed in the chromatograms of diglycosyl-GDGTs and diglycosyl-hydroxyl-GDGTs [M + H]^+^ ions (Supplementary Figure 5). These hinted at the existence of type I and type II structural isomers for most of the diglycosidic GDGTs. However, only one of the isomeric peaks was observed in the chromatograms [M + NH_4_]^+^ and [M + Na]^+^ adducts (Supplementary Figures 7, 8). This contraction suggested that there was insufficient evidence for the detection of structural isomers. 2G-GDGT-0 was an exception. The peaks corresponding to isomeric pairs were seen in the chromatograms of [M + NH_4_]^+^ and [M + Na]^+^ adducts (Supplementary Figures 6, 7). The observation of the 2G-GDGT-0 structural isomers was further accessed with MS-DIAL (Tsugawa et al., 2020) after data pre-processing (Figures 11A,B). As previously reported (Besseling et al., 2018), 2G-GDGT-0 type I and II isomers could be differentiated by their MS^2^ spectra (Figure 11C), in addition to their mobility spectra (Figure 11D).

The results are summarized in Table 2. A lipid was considered detected if at least two adducts were observed. These results were further compared to those obtained by Progenesis QI. These results suggested that manual curation had a higher identification rate than automatic data pre-processing and identification. It was because manual curation visualized the chromatograms of targeted ions and ignored the rest, whereas automatic data pre-processing required chromatographic deconvolution before spectral matching. Since some GDGTs, especially those lipids with GDGT-3 and GDGT-4 cores, cannot be chromatographically resolved, deconvolution and identification of these compounds by automation face significant challenges.

An illustration using monoglycosyl-hydroxyl-GDGTs is given in Supplementary Figures 8, 9. A summation of all the scans between 23 and 25 min revealed the isotopic envelope of 1G-OH-GDGTs (Supplementary Figure 8A). Reconstructed ion chromatograms of each of these ions resulted in four major chromatographic peaks, each of which was composed of a collection of ions (Supplementary Figure 9) forming a complex isotopic pattern (Supplementary Figures 8B–E). Interpretation of the manually curated chromatograms suggested the presence of 1G-OH-GDGT-0 to 1G-OH-GDGT-4 as their peaks were observed in the extracted ion chromatograms (Figure 10). Nevertheless, this was a convolution problem for automation. 1G-OH-GDGT-2, 1G-OH-GDGT-3, and 1G-OH-GDGT-4 were not chromatographically resolved into distinct spectral features, and only 1G-OH-GDGT-2 was identified by Progenesis QI due to its relatively high abundance.

### The Enigma of Crenarchaeol and Its Stereoisomer

Crenarchaeol and its stereoisomer (diastereomer and/or enantiomer) have been detected in marine sediment and suspected particles in seawater and their concentrations in the water column are the basis of the TEX_86_ paleotemperature proxy (Inglis and Tierney, 2020). On the other hand, recent studies on the cell cultures have revealed that the TEX_86_ signal is not dependent on growth temperature, but rather the rate of ammonia oxidation and so the energy flux of the archaeal cells (Qin et al., 2015; Hurley et al., 2016; Zhou et al., 2020). There are also questions as to their biosynthesis. Previous studies of *N. maritimus* have also come to a set of contrasting results. In an early study (Schouten et al., 2008), the regioisomer of crenarchaeol was not detected from the untreated cellular extract. Small amounts of crenarchaeol regioisomer were only detected after acid hydrolysis. Elling et al. (2014) in contrast, reported that both crenarchaeol and its regioisomer were detected as glycosidic and core lipids in the cultures of *N. maritimus*. A crenarchaeol core lipid contains one cyclohexane and four cyclopentane rings, though the stereotropic arrangement of crenarchaeol and its regioisomer has not been entirely clear (Sinninghe Damsté et al., 2018). Attempts have been made to synthesis the pair of isomers by classical chemistry, and as a result, a revised structure has been proposed (Holzheimer et al., 2020).

**FIGURE 12:**
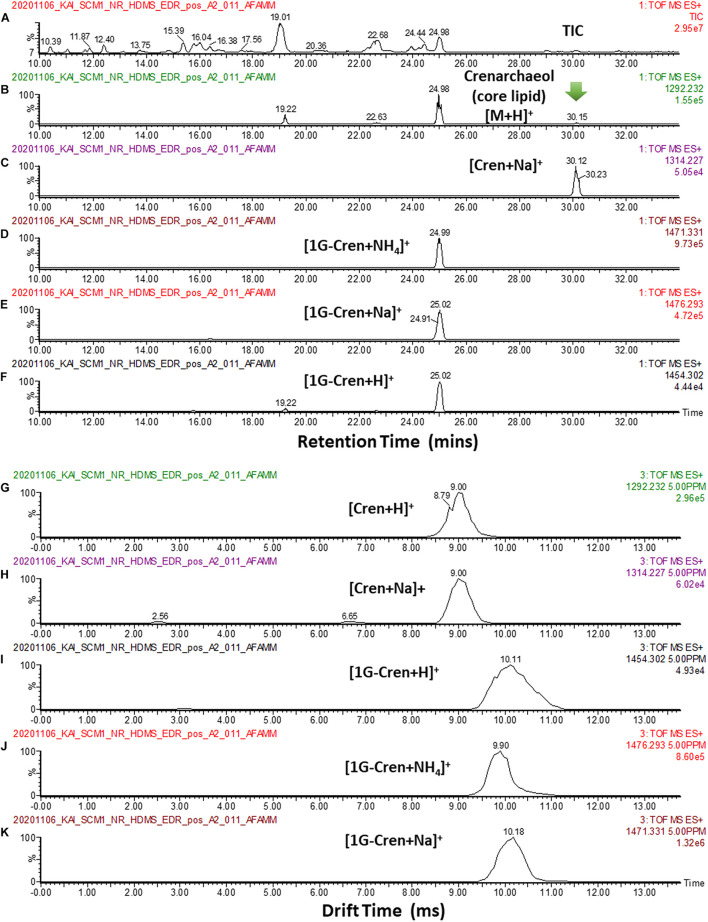
**(A)** TIC of a typical cellular lipid extract of *N. maritimus*. **(B,C)** EIC of *m/z* 1292.232, and 1314.227 corresponding to the [M + H]^+^ and [M + Na]^+^ of core crenarchaeol ion eluted at 30.15 mins. Peaks eluted at 19.22 22.63 and 24.98 mins were fragment ions of diglycosidic and monoglycosidic crenarchaeols, with the same *m/z* of the core crenarchaeol ion. **(D–F)** EIC of *m/z* 1454.302, 1471.331, 1476.293 corresponding to the [M + H]^+^, [M + NH_4_]^+^ and [M + Na]^+^ of monoglycosidic crenarchaeol. **(G,H)** Arrival time distributions of [M + H]^+^ and [M + Na]^+^ of core crenarchaeol. **(I–K)** Arrival time distributions of [M + H]^+^, [M + NH_4_]^+^ and [M + Na]^+^ of monoglycosidic crenarchaeol.

**FIGURE 13:**
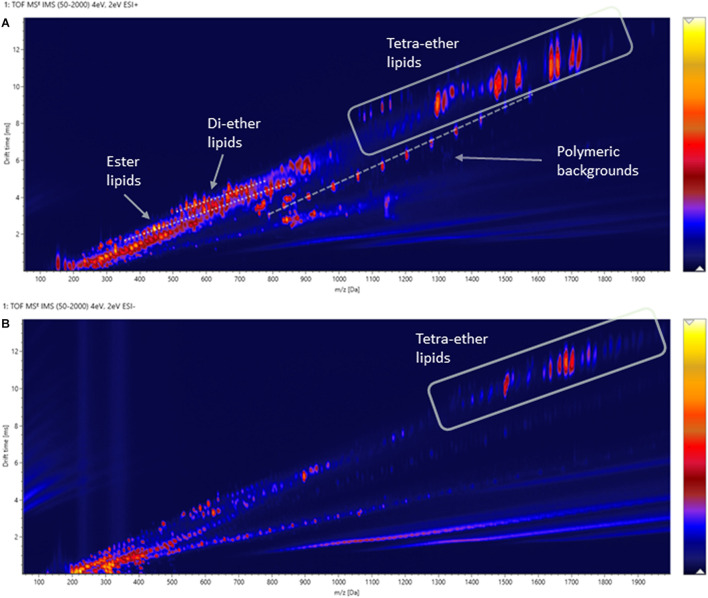
Two-dimensional IM-MS spectra (drift-time-*m/z* plot) of an experimental cellular lipid extract acquired in **(A)** positive and **(B)** negative ionization modes.

In our analysis of the cellular lipid extracts (Figure 12A), we did not observe a chromatographic separation of crenarchaeol and its proposed isomer (even as tailings), either as the core lipid (Figures 12B,C) or the monoglycosidic form of crenarchaeol (Figures 12D–F). Similarly, a single peak was observed in the ion mobility spectrum corresponding to the core lipids (Figures 12G,H), and the monoglycosidic crenarchaeol (Figures 12I–K). Our current data was inconclusive to provide further insight. While the IMS resolving power of the SYNAPT G2 class systems was inadequate for chiral discrimination, the IMS resolving power of the system permitted resolving isomeric compounds of distinct chemical classes, such as lipids from non-lipid background interferences, or differentiation of lipid subclasses, including diether and tetraether lipids of archaea from the bacterial ester lipids, based on their IM characteristics (Figure 13).

## Conclusion

Archaeal isoprenoid ether lipids were first characterized by gas chromatography-mass spectrometry (GC-MS) (de Rosa et al., 1976, 1977). The method had since become a central technique to study Archaea and their membrane lipids (Tornabene and Langworthy, 1979). However, since GC-MS requires the analytes to be volatile, the technique only could be used to characterize isoprenoid diether lipids or the phytanyl hydrocarbon chains of the tetraether lipids after chemical degradation (Chappe et al., 1980). For the next two decades, few technological advances were applied to archaeal lipid analysis until the first report of direct analysis of core lipids by HPLC-MS (Hopmans et al., 2000). The proposed method was performed on a low-cost single quadrupole system (Schouten et al., 2002). Subsequently, an improved approach made use of an ion trap system (Sturt et al., 2004; Knappy et al., 2011). The confidence of lipid identification was enhanced with multiple-stage MS^*n*^ experiments. Recent studies have gradually shifted from relative quantification of core lipids to semi-targeted fingerprinting of the lipidome using HPLC coupled to high-resolution MS (Zhu C. et al., 2013; Hopmans et al., 2016). Other techniques such as matrix-assisted laser desorption/ionization mass spectrometry (MALDI-MS) (Lobasso et al., 2015) and shotgun lipidomics (Jensen et al., 2015) have also been reported. Applications of lipidomics in geomicrobiology have already drastically advanced our understanding of the physiology of many extremophilic as well as mesophilic archaea that perform key biogeochemical processes (Law and Zhang, 2019; Law et al., 2020). However, comprehensive characterization of the lipidome remains analytically challenging, particularly the occurrence of isobaric lipid species that requires a lengthy pre-analysis fractionation or tandem chromatographic separations. With the emerging of the IM-MS technology, it is timely to explore the recent technological advances to obtain further insight into the chemical nature of archaeal lipids and to reexamine the lipidome of archaea.

A unique feature of IM-MS data is the existence of empirical correlation trendlines corresponding to chemical classes. These trendlines reflect the specific conformation of a class of compounds undertakes within the gas-phase environment of the IM spectrometer. Since gas-phase packing for lipids is inefficient, this results in a relatively large size-to-mass ratio and this allows lipids to be readily differentiated from other molecules within a two-dimensional IM-MS spectrum. Subclasses of lipids may also be differentiated by their ion mobility characteristics. For example, sphingolipids adopt larger gas-phase conformations than phospholipids because of the constraint imposed by the *sn1* sphingosine backbone that limits the degrees of unsaturation on their hydrocarbon chain (Harris et al., 2019). Similarly, the degree of cyclization equally plays a significant role in the conformation of archaeal tetraether lipids. We observed a linear increase in mobility (reduction in CCS values) with the number of cyclopentane rings in our analysis of β-L-gulopyranosyl-caldarchaetidyl-glycerols and the gradients of their correlation trendlines were a lot larger than polyalanine, PEs, PEGs and UltraMark (Figure 7). However, individual GDGT lipids were poorly separated by reverse-phase chromatography because of their minor differences in hydrophobicity (Figure 4).

Differentiation of archaeal tetraether lipid isomers was, however, less successful with ion mobility separation. While typical eukaryotic or bacterial diester lipids that differ in their backbone, headgroup, or fatty acyl composition lead to distinct structures that are resolvable in the ion mobility dimension, archaeal lipids of *N. maritimus* are not as structurally diverse as eukaryotic or bacterial lipids. Chemical diversity archetypally arises from a reorganization or arrangement of a limited number of core lipids and headgroups (Figure 1). As a result, the differences in CCS values were too small to be resolved with the SYNAPT G2 class instrumentation. Discrimination of lipid isomers, therefore, required a chromatographic separation before IM-MS measurement. This was demonstrated with the detection of isomeric lipids such as diglycosyl-dihydroxyl-GDGT-2 (Supplementary Table 1) and diglycosidic GDGT-0 (Figure 11) in our analysis of the cellular extracts of *N. maritimus*. The structural arrangement of the two hexoses led to a significant change in their relative hydrophilicity, but only a relatively small change in their CCS values. IMS could only function auxiliary in the discrimination of isomers here. Consequently, profiling of the lipidome of *N. maritim*us with HDMS*^*E*^* mode did not detect more archaeal lipids than the conventional data-dependent acquisition (DDA) approach. Indeed, our relatively rigorous approach in annotating the spectral features based on accurate masses, isotope patterns, and MS^2^ spectra against our lipid databases resulted in fewer GDGT lipids being detected by Progenesis QI than manual curation. However, our approach might have been more suited in detecting novel or unpredicted compounds, including phosphate- and/or sulfate-containing lipids in negative analysis. It is also important to stress that the implementation of an automatic data pre-processing and analysis pipeline is still advantageous and has potential applications to the analysis of other marine plankton, such as cyanobacteria and microalgae.

A major challenge of this work was the absence of an archaeal lipid database to compare the lipidome of a variety of marine archaeal species. To construct a domain-wide archaeal lipids structural library, which contained not only the reported lipids of *N. maritimus* but all known lipids produced by the domain of Archaea, a large volume of literature was reviewed. This library was essential as few reference lipids of archaea were commercially available for our method development or experimental verification. As a result, an archaeal lipid structural library in SDF format was constructed. After painstakingly constructing a structural library, a spectral library was assembled on analyzing representative cultures of marine archaea. Unfortunately, because of our inability to cultivate marine archaea and the availability of viable archaeal isolates, only a model marine *Thaumarchaeoton* was studied. This led us to generate an HDMS*^*E*^* archaeal lipid MS^2^ spectral library. The MS^2^ library was made available in an open MSP format and can be used directly with open data pre-processing software such as MS-DIAL (Tsugawa et al., 2020; Supplementary Figure 10). We have previously reviewed a selection of software packages designed specifically for lipidomic data pre-processing and analysis (Law and Zhang, 2019). To advance the field of marine microbiology, readers are encouraged to explore the available bioinformatic tools, which have different design philosophies and compatibilities with mass spectrometry systems. The structural and spectral libraries in the Supplementary Information are available to the community.

## Data Availability Statement

The data presented in the study are deposited in the MetaboLights repository, accession number MTBLS3714.

## Author Contributions

KL designed the study, conducted the investigation and analysis, developed the MS-based analytical methodology, performed data curation and validation, and wrote the manuscript. JT and WH prepared the cell cultures, performed the sample preparation, conducted the biochemical assays, and performed other administrative duties. CZ supervised the study, led the project administration, managed the resources, and reviewed and edited the manuscript multiple times before its submission. All authors approved the submitted version.

## Conflict of Interest

A patent application related to this research work is pending. The authors declare that the research was conducted in the absence of any commercial or financial relationships that could be construed as a potential conflict of interest.

## Publisher’s Note

All claims expressed in this article are solely those of the authors and do not necessarily represent those of their affiliated organizations, or those of the publisher, the editors and the reviewers. Any product that may be evaluated in this article, or claim that may be made by its manufacturer, is not guaranteed or endorsed by the publisher.

## Footnotes

1 see later in Figure 13.

## Supplementary Material

Supplementary Table 1 List of detected lipids and their CCS values.

Archaeal Lipid Structural Library (in SDF format).

Archaeal Lipid HDMS*^*E*^* MS^2^ Spectral Library (in MSP format).
