# Supplementary material for: A Novel Approach to Characterize the Lipidome of Marine Archaeon Nitrosopumilus maritimus by Ion Mobility Mass Spectrometry
Source: Front Microbiol. 2021 Dec 2;12:735878. doi: 10.3389/fmicb.2021.735878 (PMC8674956; doi:10.3389/fmicb.2021.735878)
Supplement: Supplementary file 2 [file Table_1.DOCX]

Supplementary Material

**A**


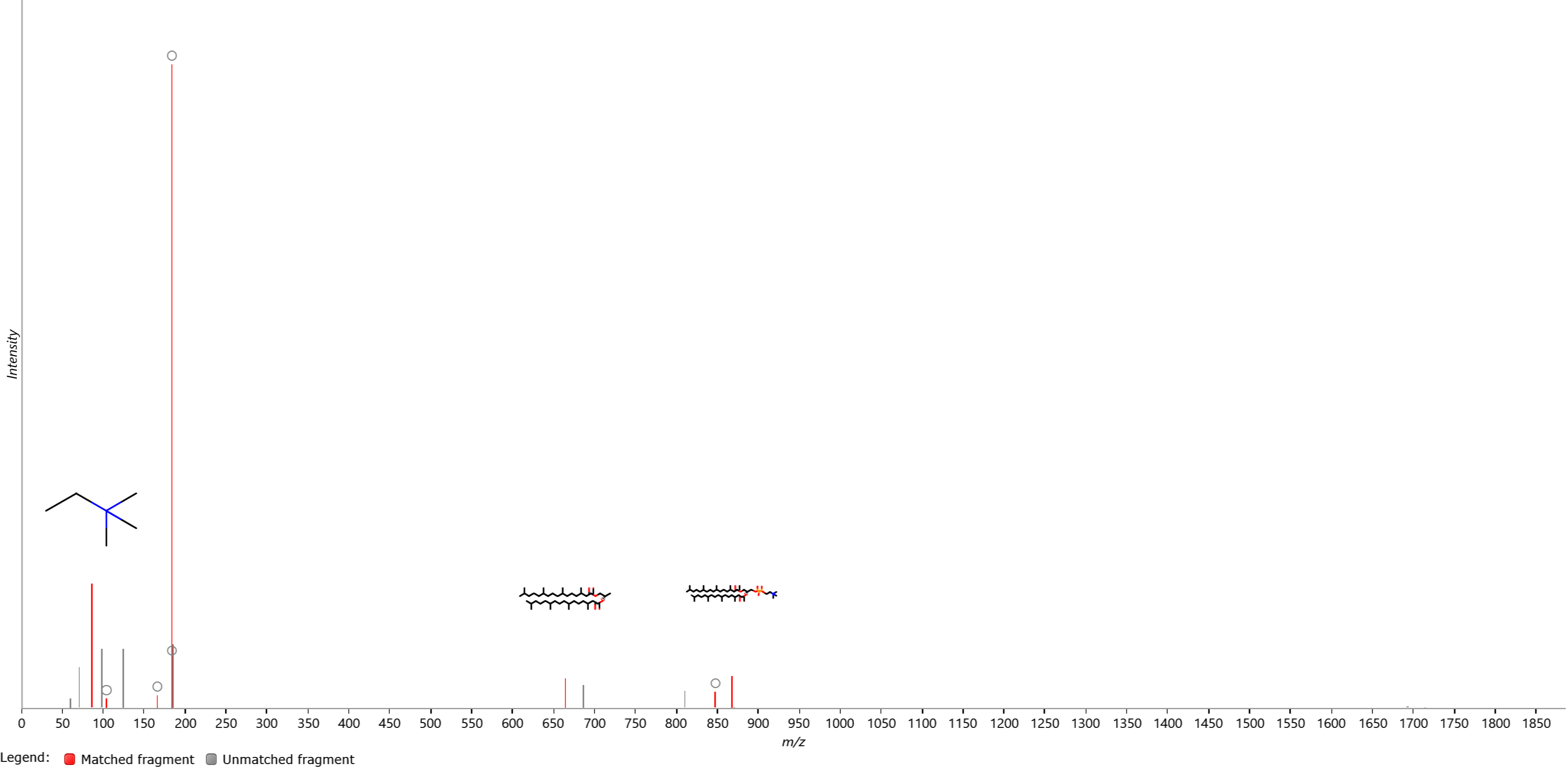


**Fragmentation trace of 20:0 PC**

**Fragmentation trace of 4ME 16:0 PC**


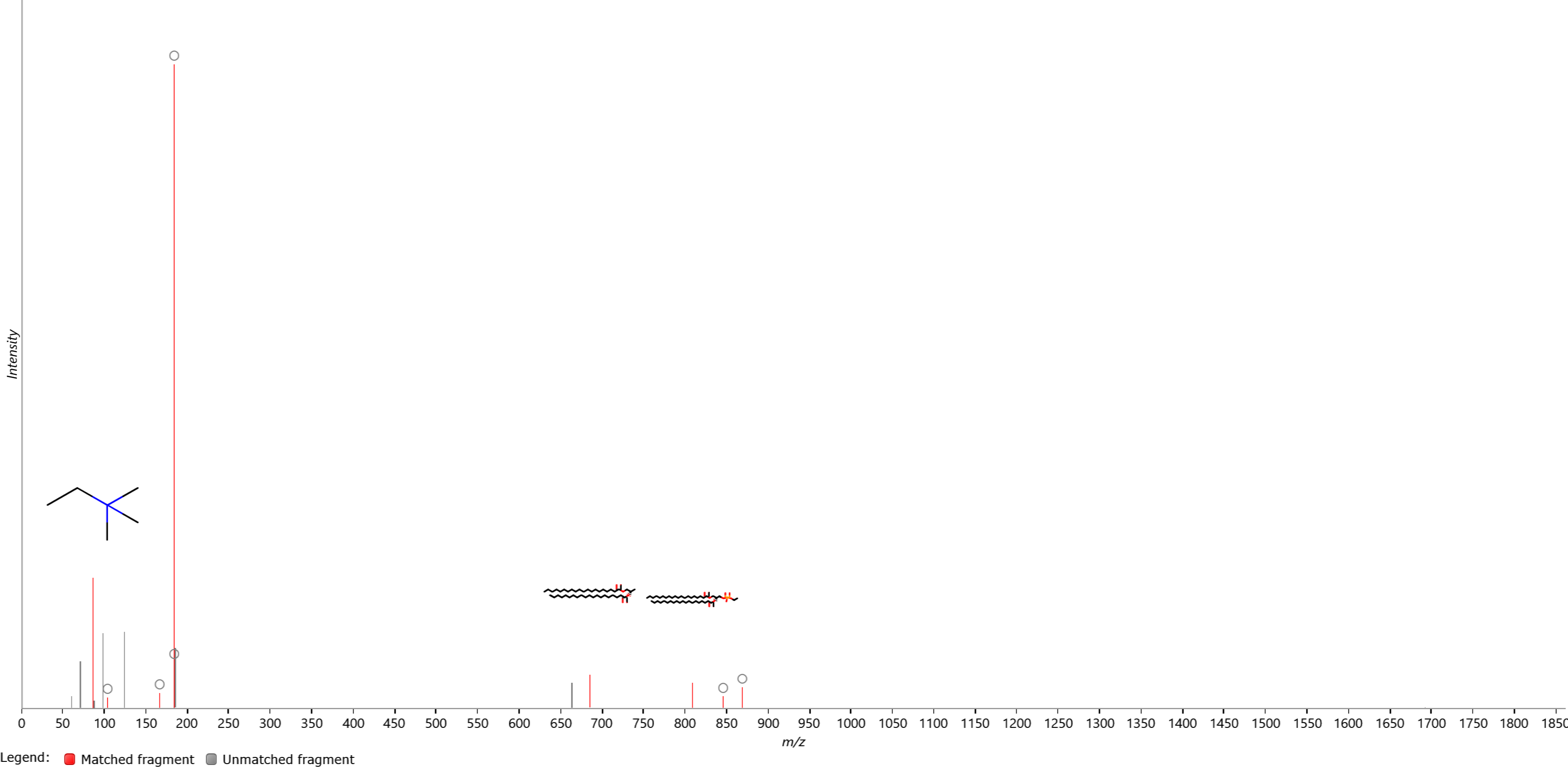


**B**

**Supplementary Figure 1.** HDMS*^E^* MS^2^ mass spectra of the isomeric lipid pair: (A) 4ME 16:0 PC and (B) 20:0 PC. Fragment ions consistent with the *in-silico* fragmentation were highlighted.


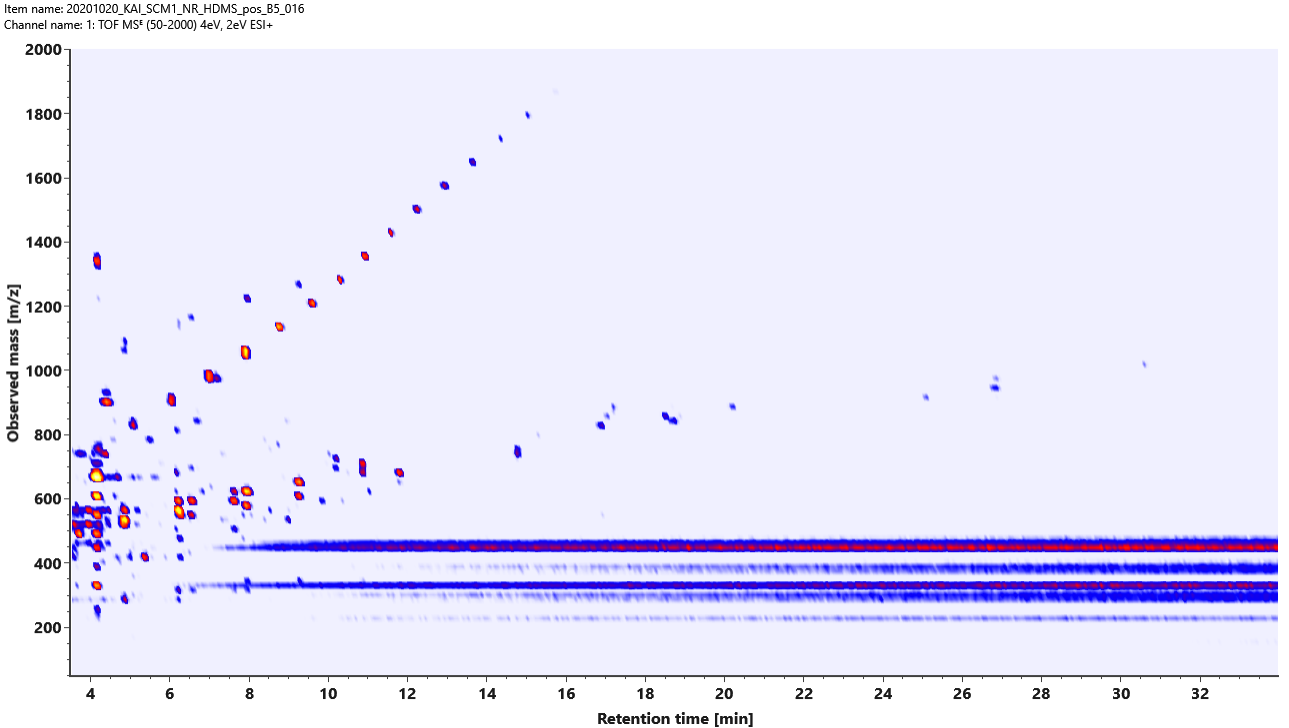


Polymeric backgrounds


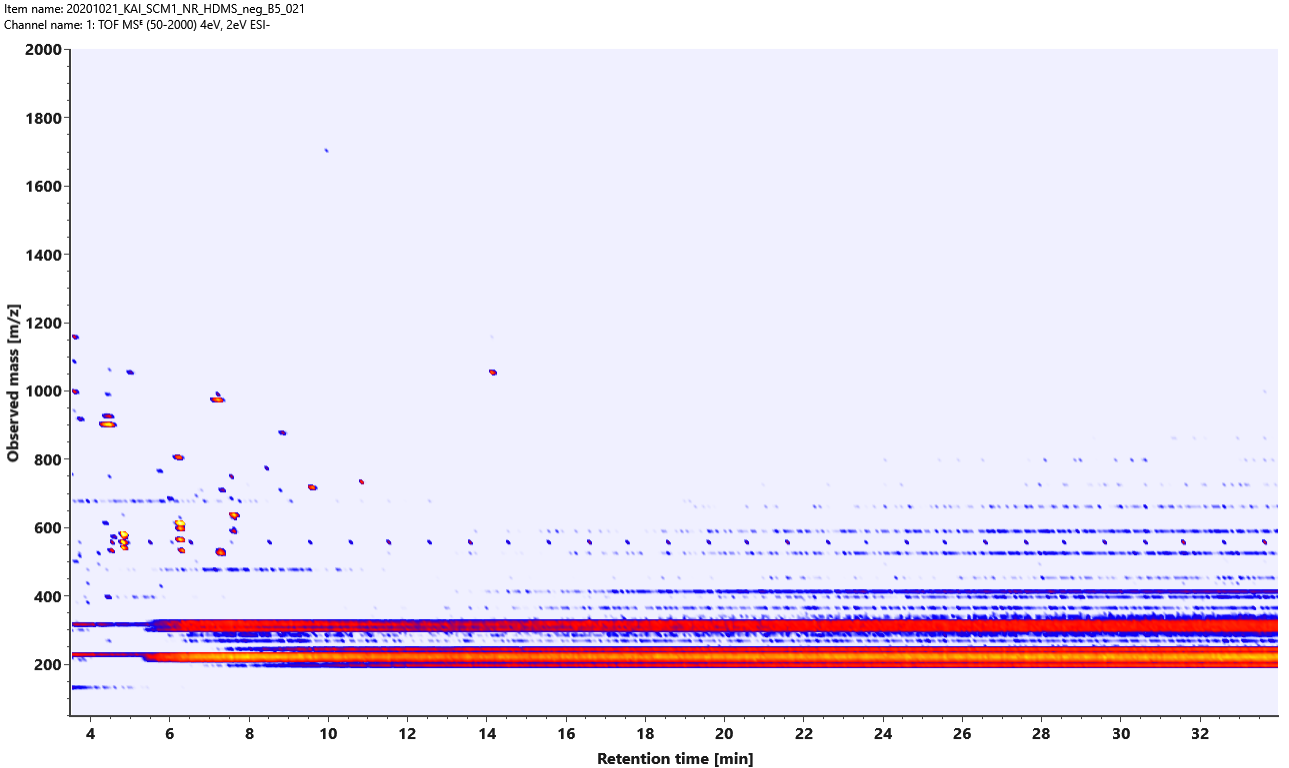


**Supplementary Figure 2.** Density maps (*rt-mz* plots) of a cell-free control acquired in (A) positive and (B) negative ionization modes.

?

Set 1

Set 2

**Supplementary Figure 3.** Extracted ion chromatographs of 1G-GDGTs (top) and 1G-OH-GDGTs (bottom) [M+NH_4_]^+^ ions.

Set 1

Set 2

**Supplementary Figure 4.** Extracted ion chromatographs of 1G-GDGTs (top) and 1G-OH-GDGTs (bottom) [M+Na]^+^ ions.

**Supplementary Figure 5.** Extracted ion chromatographs of 2G-GDGTs (top) and 2G-OH-GDGTs (bottom) [M+H]^+^ ions.

**Supplementary Figure 6.** Extracted ion chromatographs of 2G-GDGTs (top) and 2G-OH-GDGTs (bottom) [M+NH_4_]^+^ ions.

**Supplementary Figure 7.** Extracted ion chromatographs of 2G-GDGTs (top) and 2G-OH-GDGTs (bottom) [M+Na]^+^ ions.

C

D

E

B

A

Summation of the scans between 23-25 mins (all the peaks)

**Peak 3**

**Peak 2**

**Summation of the scans of Peak 4**

**Summation of the scans of Peak 1**

**Supplementary Figure 8.** (A) Summation of the scans between 23-25 mins where 1G-OH-GDGTs were eluted. Protonated ions 1G-OH-GDGTs and isotopic peaks were seen in between *m/z* 1470-1490. (B-E) Summation of the scans of the chromatographic peaks 1-4 in Supplementary Figure 9. Each reconstructed chromatographic peak therefore contained a mixture of multiple ions. Ion peaks labelled with arrows corresponding to 1G-OH-GDGTs. Isotopic peaks of 1G-OH-GDGT-4 (labeled with blue arrows in (b)) overlapped with the molecular ion peaks of 1G-OH-GDGT-3 and 1G-OH-GDGT-2.

1G-OH-GDGT-2

1G-OH-GDGT-3

1G-OH-GDGT-4

**Peak 3:** 1484, 1483, 1482,

1479, 1478

**Peak 2:** 1486, 1485, 1484,

1481, 1480, 1479

1G-OH-GDGT-0

**Manual interpretation**

**Peak 4:** 1477, 1476,

1474, 1473, 1972, 1971

**Peak 1:** 1489, 1488, 1487, 1486, 1481

Not identified by automated processes

1G-OH-GDGT-1

**Supplementary Figure 9.** Reconstructed ion chromatogram of the ion peaks in Supplementary Figure 8(a). Four major chromatographic peaks were observed and were labeled as Peak 1 to 4. Each chromatographic peak was a collection of a mixture of protonated ions and isotopic peaks of two or more GDGTs. Manual interpretation of the chromatogram indictaed that 1G-OH-GDGT-0 was in peak 2, 1G-OH-GDGT-1 was in peak 3. 1G-OH-GDGT-2 to 1G-OH-GDGT-4 were co-eluted and were in peak 4.


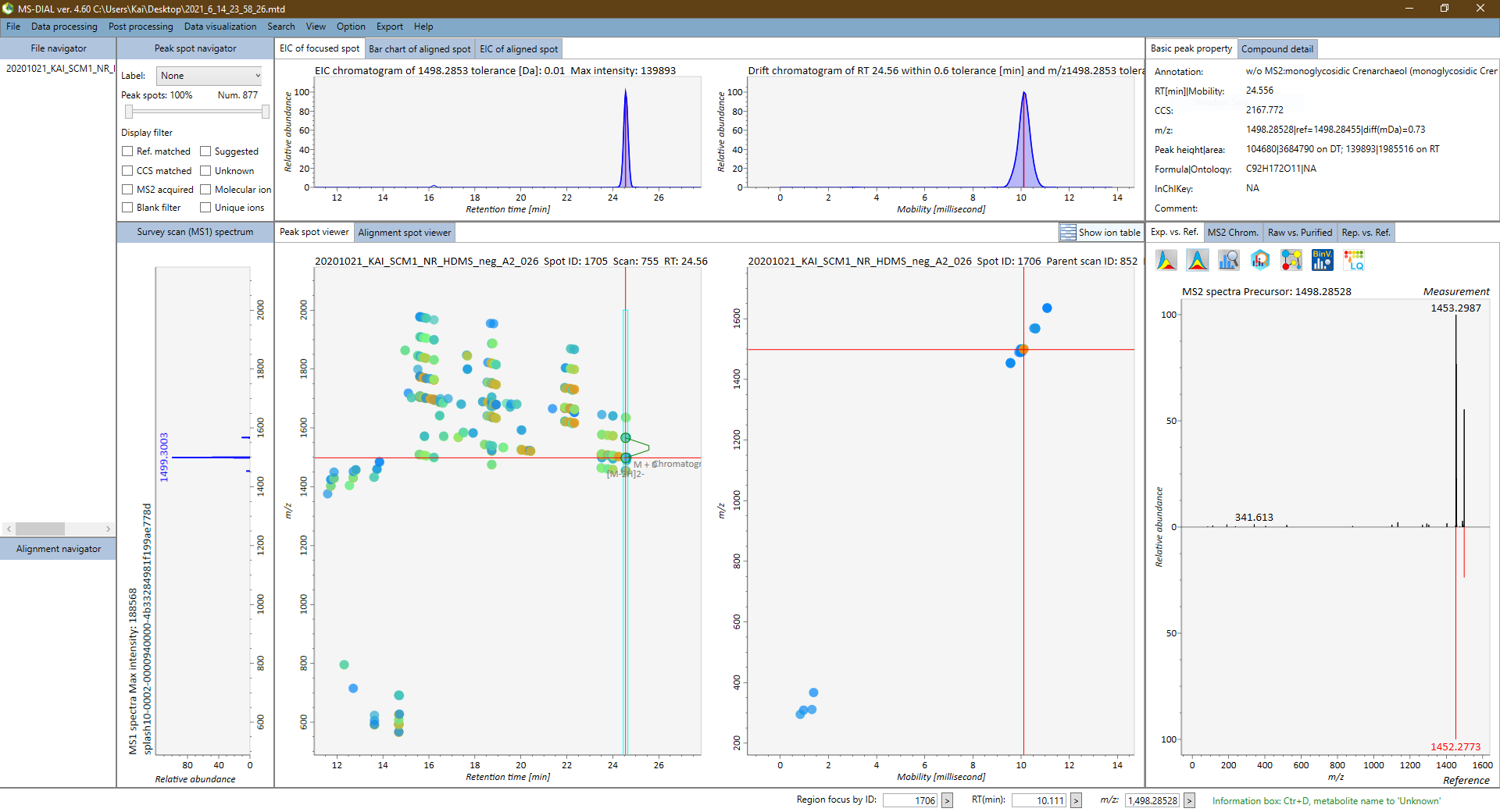


**Supplementary Figure 10**. Raw TWIMS-MS data acquired by MasssLynx and the MSP spectral library created by Progenesis QI in this work can be used with MS-DIAL. The data shown were acquired in negative ionization mode.
